# The Integration of Artificial Intelligence Into Patient Care: A Case of Atrial Fibrillation Caught by a Smartwatch

**DOI:** 10.7759/cureus.35941

**Published:** 2023-03-09

**Authors:** Angad Bedi, Mohammad Khaldoun Al Masri, Hussam Al Hennawi, Shayan Qadir, Patrick Ottman

**Affiliations:** 1 Internal Medicine, Abington Jefferson Hospital, Abington, USA; 2 College of Medicine, Alfaisal University College of Medicine, Riyadh, SAU

**Keywords:** holiday heart syndrome, arrythmias, palpitations, artificial intelligence in medicine, smart watch, atrial fibrillation

## Abstract

Artificial intelligence (AI) offers a wide range of applications in clinical practice, and new technologies are rapidly evolving the healthcare industry and enhancing outcomes. Smartwatches represent the most popular type of wearable AI device that can assist people in detecting cardiac arrhythmias via constant monitoring of heart activity. Numerous advantages result from integrating AI into healthcare systems, including improved patient care, lower rates of medical errors, better treatment recommendations, and more accurate diagnosis of diseases. However, doubts still remain regarding the adoption of AI into patient care due to the challenges it poses. In this paper, we report a case of atrial fibrillation (AF) in a young patient that was detected by his smartwatch. We also highlight some of the benefits and challenges of AI applications in healthcare.

## Introduction

Artificial intelligence (AI) refers to the ability of a computer system to imitate different aspects of human intelligence, such as reasoning, learning, and problem-solving, in an autonomous fashion without human intervention. The use of advanced AI technologies has become increasingly widespread across various industries, including retail, manufacturing, supply chain, finance, and healthcare, with resulting substantial impacts on performance and productivity [1]. Today, AI techniques like natural language processing (NLP) and algorithmic machine learning are being largely adopted in clinical practice, allowing healthcare professionals to accurately diagnose diseases and provide tailored treatment plans and more efficient follow-up care [2]. On the other hand, tech-savvy consumers have been leveraging AI-based wearable gadgets over the past decade to keep track of their health, lifestyle habits, and fitness level, rendering themselves more capable of monitoring their own physical well-being.

Smartwatches are a form of modern wearable AI technology that empowers users by promoting self-care through constant measurement of health metrics such as vital signs, body mass index, and blood glucose, among many others. The powerful features and flexibility they offer play a major role in facilitating the development of patient-centric healthcare approaches [2]. However, it remains unclear whether such devices can be reliably integrated into patient care.

## Case presentation

A 25-year-old male with a past medical history of anxiety and depression presented to the emergency department with a complaint of pressure-like chest discomfort and palpitations for the last 24 hours. The patient sought medical attention after his smartwatch notified him several times of an irregular heart rhythm consistent with atrial fibrillation (AF), likely triggered by an episode of binge drinking over the weekend and possibly resulting in holiday heart syndrome. He denied cough, dizziness, focal weakness, numbness/tingling, fever, night sweats, and other constitutional symptoms, but noted a slight shortness of breath on exertion. His family history was negative for inherited arrhythmias or sudden cardiac death, although he stated that he had annual follow-ups as a child for an aortic valve defect until he was medically cleared at the age of 18. The patient denied losing consciousness during activity or experiencing similar symptoms previously. He admitted to tobacco and occasional social alcohol use but denied any use of illicit drugs.

**Figure 1:**
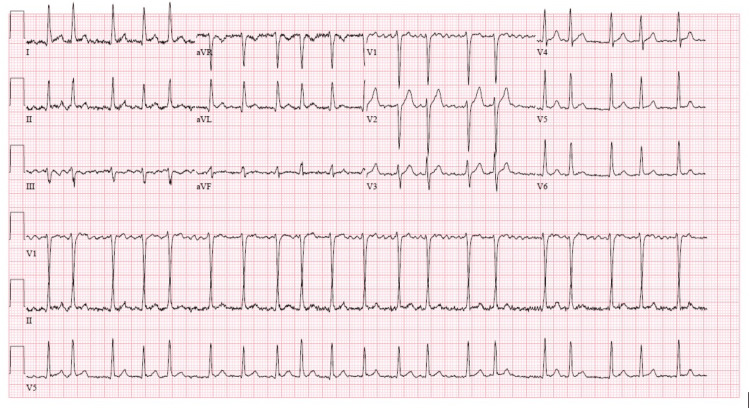
Electrocardiogram demonstrating atrial fibrillation with rapid ventricular response at a rate of 123 bpm. No ischemic changes or abnormal cardiac axis are seen.

Initial investigation in the emergency department showed that the patient was tachycardic with rates consistently above 120 bpm but otherwise vitally stable, and an electrocardiogram (ECG) revealed atrial fibrillation with a rapid ventricular response (Figure 1). He was given intravenous Diltiazem and oral metoprolol for rate control, but without resolution. Therapeutic subcutaneous Enoxaparin was also started. Further workup was performed, including serial troponin, D-dimer, INR, complete blood count, comprehensive metabolic panel, magnesium, thyroid stimulating hormone, urinalysis, and urine and serum drug screening, which was unremarkable. However, an elevated brain natriuretic peptide (BNP) of 796 was noted, but a subsequent chest X-ray showed no evidence of heart failure or acute disease. The patient was admitted for further evaluation and monitoring, and a cardiology consultation was requested. Following admission, the Diltiazem drip was resumed to aid in rate control.

Due to the resultant hypotension, the diltiazem infusion was held for a short period of time and then restarted at a lower dose to avoid any further drops in blood pressure. The patient continued to remain in AF, which was rate-controlled. Upon the cardiologist's recommendation, the patient underwent transesophageal echocardiography (TEE)-guided electrical cardioversion the next day, and normal sinus rhythm was successfully restored. Consequently, the patient became medically stable for discharge, and despite having a CHA2DS2-VASc score of 0, anticoagulation with Apixaban was recommended for four weeks as a prophylaxis for thromboembolic events. Alcohol cessation was also discussed with the patient to preclude possible exciting events that may trigger further episodes of AF. Additionally, he was advised to limit heavy exercise with a slow and gradual increase in the amount of activity. Monthly cardiology follow-up visits were recommended for the patient to monitor his condition instead of solely relying on his smartwatch.

## Discussion

Healthcare systems nowadays are burdened by large amounts of wasted resources, be it the high costs of unnecessary tests and treatments, false-positive diagnoses of diseases, or poorly designed clinical trials. Patients equally endure the consequences at times as a result of unreported medical errors and adverse events made by healthcare professionals, often impacting their safety and decreasing overall satisfaction with the care provided [3,4]. Nevertheless, recently introduced AI-powered technologies can process tremendous amounts of data faster and more accurately than their human counterparts, consequently leading to significant advantages in the medical and scientific fields [2]. Integrating AI into healthcare systems allows for a wide range of benefits, including greater accuracy in diagnosis, reduced rates of medical errors, better treatment recommendations, enhanced patient care, and more efficient administrative workflow [5,6]. Cost savings is another noteworthy factor that encourages AI implementation in healthcare, as a 2017 analysis by Accenture found that AI applications can potentially cut up to 150 billion dollars of annual U.S. healthcare expenses by 2026 [7].

Clinical applications of AI are vast and diverse, with innovations rapidly transforming the health industry and improving outcomes. A study done in China reported that colonoscopies performed with the assistance of an AI system had higher detection rates of adenomas and small polyps compared to conventional colonoscopy screening [8], thereby allowing for the early management of possibly cancerous lesions. Pesapane et al. [9] explained how AI will augment radiologists' efforts through machine and deep learning, where smart computers can analyze and interpret more medical images with much superior accuracy than specialists would alone, thus facilitating better clinical decision-making. Natural language processing techniques have been shown to be promising in identifying suspected Alzheimer's disease by performing linguistic analysis of short samples of patients' speech and language [10].

Smartwatches are a prevalent example of AI technology that utilizes pulse waves and deep neural network (DNN) algorithms through optical sensors for real-time recording and analysis of heart rate and rhythm [3,11]. They can help individuals with diagnosed and undiagnosed arrhythmias like atrial fibrillation by continuously monitoring their vital signs and alerting them when irregular heart activity emerges. AF is the most common type of cardiac arrhythmia seen in clinical practice [12] and is projected to affect more than 10 million people in the U.S. by 2050 [13]. With the widespread growth in the use of wrist-worn devices, several reports on their ability to identify new episodes of AF are coming to the surface. The Apple Heart Study in 2019 [14] revealed that 34% of individuals who were alerted by their Apple watches about an irregular rhythm were later diagnosed with AF, and the positive predictive value in subjects notified of an irregular pulse was 84%. However, these observations were based on the assessment of older generation Apple watches that can only detect an irregular pulse through optical sensors without ECG-recorded evidence of the heart rhythm, therefore relying on a separate ECG test to confirm the diagnosis of AF after receiving the initial notification on the watch. In a more recent study, Seshadri et al. [15] tested the accuracy of newer generation Apple watches, which can detect pulse abnormalities and also record irregular rhythm using built-in electrodes to create a single-lead ECG report. The study included 50 post-operative patients who had a telemetric assessment of their cardiac rhythm three times per day over two days, in addition to rhythm recording using an Apple watch. Of the 292 obtained telemetry assessments, AF was detected in 90 instances, of which 34 were correctly identified by the smartwatch with a resulting sensitivity of 41%. Despite the promising results in the current literature, further research is warranted to better determine the reliability of smartwatches in detecting and monitoring arrhythmias.

The era of digital medicine presents new opportunities for optimizing clinical practice, but several challenges need to be effectively addressed in order for AI to be successfully incorporated into healthcare. Some of the perceived obstacles to AI applications include data governance and sharing, software failure concerns, job displacement, communication barriers between patients and machines, and accountability for medical errors [16]. Furthermore, possible data breaches of patients' health information stored in virtual servers raise ethical questions about the privacy and safety surrounding the use of AI-powered systems [17]. Uncertainty remains high about the potential of AI integration in healthcare, but its benefits will be reaped for many decades once these challenges are overcome.

## Conclusions

Our case exhibits how the application of artificial intelligence technology could aid in patient care, where the patient was notified by his smartwatch about an irregular heartbeat for which he sought medical attention and was later diagnosed with atrial fibrillation. Given the importance of early detection, AI-powered devices can be of great value in managing AF and its life-threatening consequences. There are many advantages to integrating AI into healthcare systems, but in order to do so, several ethical and technical challenges need to be tackled.
